# From state to change of state by type-shift

**DOI:** 10.1007/s11050-025-09234-x

**Published:** 2025-05-28

**Authors:** Ryan Walter Smith, Jens Hopperdietzel, Andrew Koontz-Garboden

**Affiliations:** 1 Department of Linguistics, Ohio State University, Columbus, OH USA; 2 Department of German Language and Literature I, University of Cologne, Cologne, Germany; 3 Department of Linguistics and English Language, University of Manchester, Manchester, UK

**Keywords:** Change of state, Type-shifting, Lability, Blocking, Structural alternatives

## Abstract

This paper proposes a *type-shifting* analysis of *state/change-of-state lability* (Koontz-Garboden et al. in Verbhood and state/change of state lability across languages, Ms., 2023): in languages with no overt inchoative morphology, a type-shifting operation, which we dub *Inchoative Shift*, introduces inchoative semantics where type mismatches would otherwise occur. In combination with a Blocking Principle, drawing on Chierchia’s (Natural Language Semantics 6:339–405, 1998) proposal, the analysis explains the restricted distribution of change-of-state meaning in labile languages, and the fact that change-of-state readings of stative predicates are in complementary distribution with inchoative morphology cross-linguistically. Furthermore, once we augment the Blocking Principle with a notion of structural alternatives (Katzir in Linguistics and Philosophy 30:669–690, 2007), we can provide an explanation for the fact that the presence of *periphrastic* inchoative constructions does *not* always block inchoative type-shifting in a language. Our account improves on previous approaches to state/change-of-state lability, such as Koontz-Garboden’s (Journal of Linguistics 43(1):115–152, 2007) *coercion* analysis, by virtue of making broad and, as we argue, correct predictions about the distribution of state/change-of-state lability and its interaction with other change-of-state expressions cross-linguistically.

## Introduction: state/change-of-state lability

In the context of property concept lexemes (words that serve as the translational equivalents of English adjectives, as in Dixon 1982; Thompson 1989, henceforth PCLs), three derivational relationships can be identified between words describing a state and words describing changes into that state. The first type is *equipollent*: both stative and change of state are equally derived from a common root, e.g., in Ulwa (Misumalpan; Hale and Keyser 2002; Koontz-Garboden 2005).

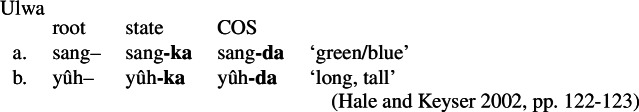

In the second type, the stative form is morphophonologically less marked than the change of state form, as in English, Yup’ik (Eskimo-Aleut), or Warlpiri (Pama-Nyungan).

(2)

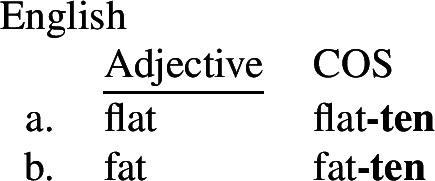

(3)

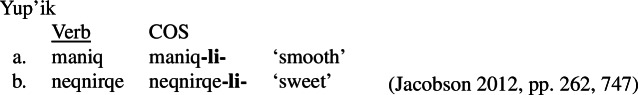

(4)
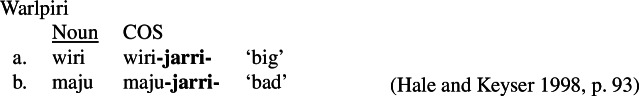

The third derivational type, and our focus in this paper, is *labile*, in which there is no surface morphophonological difference between state and change of state lexemes, as illustrated by Daakaka (Oceanic), Paragyuan Guaraní (Tupi) and Mandarin (Sino-Tibetan) below (Hopperdietzel 2021; Califa 2018; Zhang 2023; see Koontz-Garboden et al. 2023 for an overview).^1^

(5)

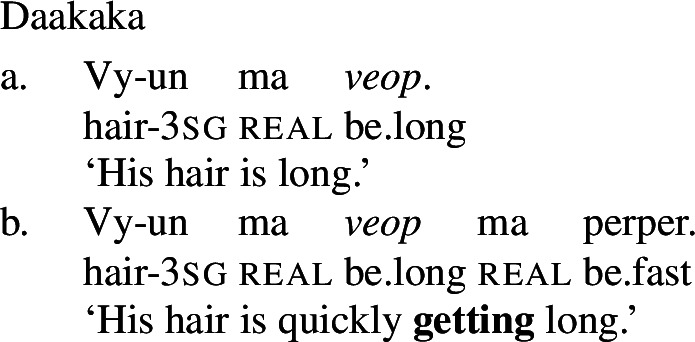

(6)

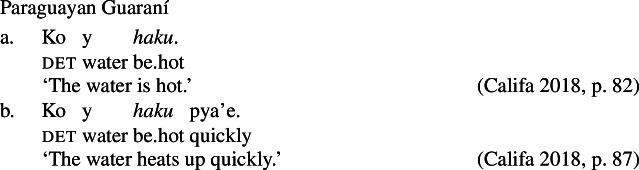

(7)
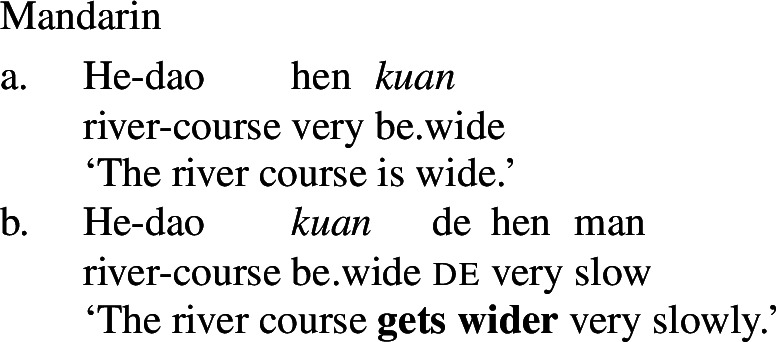

Cross-linguistically, we observe three important properties of the distribution of state/change-of-state lability. First, state/change-of-state lability is tied to verbal category: typologically, state/change-of-state lability is primarily observed in languages in which stative lexemes are predominantly of verbal category, as, for example, in Tongan, Daakaka, and Mandarin above (Bowler et al. 2022; Koontz-Garboden et al. 2023; cf. Chung and Timberlake 1985). In contrast, apparent lability is only rarely observed with nonverbal stative lexemes, e.g., English deadjectival change-of-state verbs lacking in overt marking (*the clear table* vs. *clear the table*), and exhibits different underlying properties (as discussed immediately below). This suggests that the restriction of lability to verbal property concepts is, in the general case, non-accidental, and requires a systematic explanation.

A second important property of labile languages is that the change-of-state reading of a PCL is not freely available. We can illustrate this with the examples in (7) and (7) above: the (a) sentences in these examples possess only a stative reading, and are infelicitous in contexts involving explicit change of state (cf. Koontz-Garboden 2007 on Tongan). A change-of-state reading is possible only in the presence of expressions that require eventive semantics, such as the rate adverbials in the (b) sentences above. State/change-of-state lability, as defined in this paper, must therefore be distinguished from cases of zero derivation, in which the alternation between a stative and change-of-state reading is freely available in the absence of overt morphology and event-selecting material.^2^ While this kind of covert derivation is sometimes observed in languages with nonverbal PCLs such as English *clear* above, it is also attested in languages with verbal PCLs, as illustrated by Jamul Tiipai (Yuman) below (Miller 2001).

(8)
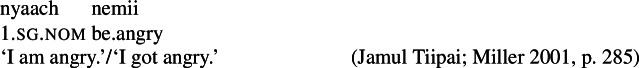

Finally, shifts from a stative to a change-of-state interpretation are not attested with stative verbs in all languages. In particular, in languages that fall into one of the non-labile classes in (1)-(4) above, even stative PCLs of verbal category do not exhibit change-of-state meaning in the presence of an event-selecting expression. For example, in Japanese, a language of the equipollent type, and Korean, a language of the derivational type, stative PCLs do not have a change-of-state meaning in the presence of a rate adverbial, and are in fact unacceptable with them. Instead, such languages make use of a change-of-state verb derivationally related to the PCL to express change of state in the presence of a rate adverbial (Fritz-Huechante et al. 2018 on Korean; see Zubizarreta and Oh 2006; Lim 2016; Choi 2015 for further discussion).

(9)

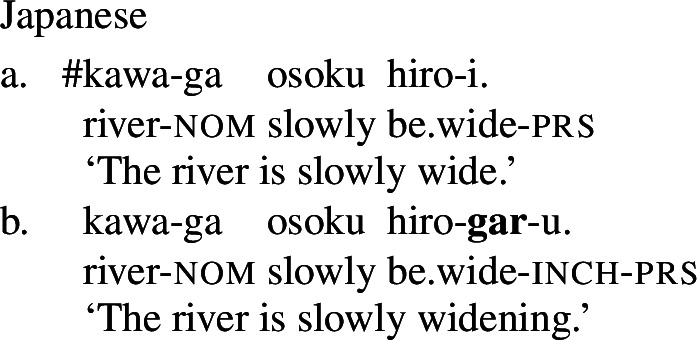

(10)
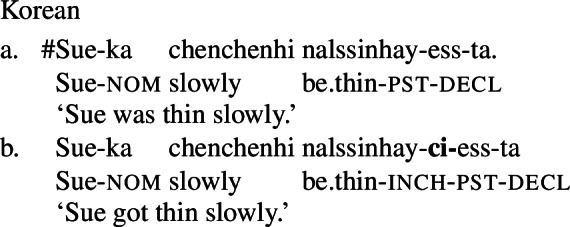

The different behavior of labile and non-labile languages suggests a relationship between the absence of overt derivational morphology and the appearance of change-of-state meaning in the presence of event-selecting expressions. We can state this generalization as follows: *the appearance of change-of-state meaning with stative lexemes in the presence of event-selecting material is in complementary distribution with the existence of derivational change-of-state morphology in a language*. An adequate theory of the labile derivational relationship must be able to derive this generalization, in addition to the other properties of lability outlined above.

The paper is structured as follows. Section 2 develops a type-shifting perspective on lability, defining an operation of Inchoative Shift that introduces inchoative semantics where type mismatches would otherwise occur, and shows how this operation provides an account of the key properties of labile languages discussed above, while making additional predictions beyond these phenomena. Section 3 develops a Generalized Blocking Principle for Inchoative Shift, building on Chierchia’s (1998) Blocking Principle for determiner-like type-shifting principles, but immediately notes a problem for the account presented by the presence of periphrastic inchoatives in languages that nevertheless permit Inchoative Shift. We then present a refinement of the Blocking Principle so that it is sensitive to structural complexity, drawing on work due to Katzir (2007) in the domain of the generation of alternatives for scalar implicature, thereby accounting for the co-occurence of Inchoative Shift and periphrastic inchoatives. Section 4 defends the type-shifting + blocking analysis from reasonable alternatives, particularly an analysis in terms of null morphology and the coercion analysis of lability in Tongan due to Koontz-Garboden (2007). Section 5 discusses potential confounds in the analysis, in particular two notions of change of state that may be relevant to the proper formulation of Inchoative Shift cross-linguistically, independently motivated readings of rate adverbs that could in principle play a role in generating inceptive readings with stative predicates (Rawlins 2013; Koev 2017), and the analysis of perfect morphemes as introducing inchoative semantics in Matthewson et al. (2015). Here, we show that, once these potential confounds are controlled for, no problem arises for our analysis. Section 6 concludes the paper with a discussion of type-shifting operations in natural language and future research.

## Lability as type-shifting

### Inchoative Shift

We propose that the non-randomness of lability with verbal property concepts requires a systematic explanation whereby with state/change-of-state relations, morphologically speaking, what you see is what you get: there is no morpheme, either overt or covert, that encodes change-of-state semantics in labile languages. Instead, state/change-of-state lability arises via a *type-shifting operation* that applies to verbal constituents denoting a predicate of states and returns a predicate of events.^3^ Specifically, the operation takes a predicate of states, existentially closes the state argument, and introduces a change-of-state relation become between an event and the state.^4^ We term this operation *Inchoative Shift* (11-a), modeled on Kratzer’s (2005) rule of Causative Shift. For the sake of concreteness, we define the become relation as in (11-b), following Beavers and Koontz-Garboden (2020).

(11)
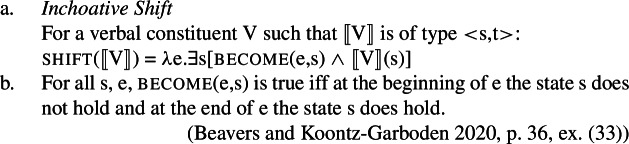

Following an idea found throughout the type-shifting literature, Inchoative Shift applies only as a *last resort* mechanism to repair local type mismatches (Partee and Rooth 1983; Bittner 1998; Chierchia 1998; Winter 2007). This property of type-shifting, we claim, is the source of the restriction of change-of-state readings with stative predicates in labile languages to cases where the VP would serve as the argument to an event modifier, such as a rate adverb in the examples above. As rate adverbs take predicates of *dynamic eventualities* as arguments (Creswell 1978; Rawlins 2013; Koev 2017), composition of a rate adverb with a predicate of states would fail, all else being equal. Type-shifting by Inchoative Shift occurs in exactly these instances, converting a predicate of states into a predicate of events, thereby allowing composition with a rate adverb to proceed. The tree in (13) provides an illustration of a Tongan VP undergoing Inchoative Shift in the presence of a rate adverb.^5^ Note that the node labeled ↑VP is merely meant as a representation of the result of applying Inchoative Shift to the denotation of the VP, and is not meant to be understood as a node distinct from VP.

(12)

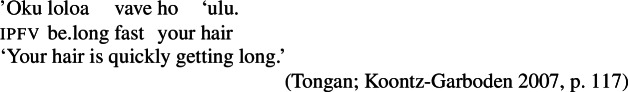

(13)

In the absence of the need to compose with a function from predicates of dynamic eventualities, no type mismatch arises. Since no type-mismatch needs to be repaired, Inchoative Shift does not apply, and we therefore predict purely stative readings with stative VPs in such situations, as is indeed the case in the (a) examples in (5-7) above. The type-shifting perspective thus offers a principled explanation for the limited distribution of change-of-state semantics in labile languages.

In the next subsections, we turn to additional predictions that our formulation of lability as Inchoative Shift makes beyond the phenomena we described at the outset.

### The generality of Inchoative Shift

As our type-shifting rule applies generally in cases where a function from event predicates needs to compose with a stative predicate, we expect change-of-state semantics to occur in more contexts than with just rate adverbials. This prediction is borne out. For example, Inchoative Shift also applies in aspectual contexts requiring the event described by the verb to be over, such as with perfect and perfective aspects, as can be seen in the Tongan, Mandarin and Daakaka examples in (14), (15), and (16), respectively (cf. Koontz-Garboden 2007; Tham 2013; also Krajinovic 2020 on Nafsan and Matthewson et al. 2015 on Niuean [both Oceanic]).^6^

(14)

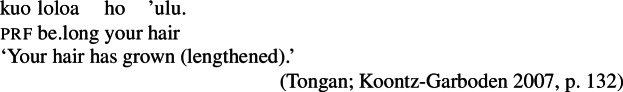

(15)

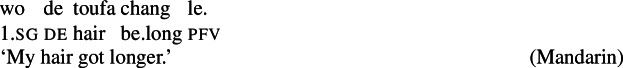

(16)
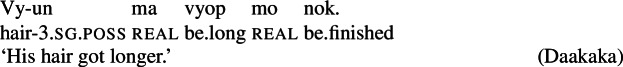

Moreover, progressive aspect, which selects for dynamic predicates, also triggers the application of Inchoative Shift, as illustrated by Daakaka below.

(17)
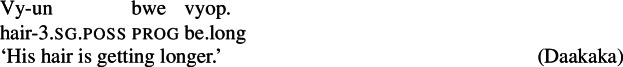

Finally, we see the same effect for predicates that subcategorize for a dynamic predicates. An example of this is the morpheme *‘osi* ‘finish’ in Tongan, which, according to Koontz-Garboden (2007, p. 134), is compatible only with expressions that denote predicates of dynamic events. When it composes with a stative predicate, *’osi* can only be used in a situation in which an event ended with an object in the state named by the stative verb, and not, for instance, in a situation in which the state in question ended by virtue of no longer holding.

(18)
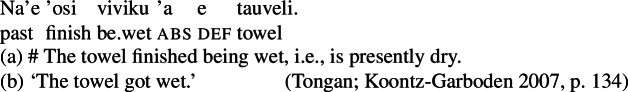

Inchoative Shift is thus not to be seen as a phenomenon observed only with particular classes of expressions, such as rate adverbs, such that its effects could be ascribed just to the semantics of that class of expressions. Rather, Inchoative Shift is a general type-shifting operation that applies in a range of cases, unified by a common requirement to compose stative and eventive material.

### Categorial restrictions

Note that we have formulated Inchoative Shift in such a way that its application is restricted to verbal constituents, i.e., verbs and VPs. This has two sources of motivation. The first source is typological: Koontz-Garboden et al. (2023) and Bowler et al. (2022) observe that, cross-linguistically, state/change-of-state lability occurs with verbs, but not with nouns or adjectives. In other words, across languages, stative verbs appear with change-of-state semantics in certain environments, but stative nouns and adjectives do not show the same shift in meaning. A restriction of Inchoative Shift to verbal constituents makes sense of this typological observation, as constituents of verbal category would be the only ones capable of undergoing the shift in the first place.

In addition to the typological evidence, we also find empirical evidence for a restriction of this kind *within* particular languages, particularly in those that possess both a class of stative verbs and a distinct nonverbal class. In such languages, only stative *verb* phrases, but not *nonverbal* phrases, may undergo Inchoative shift. We illustrate this with the Oceanic language Daakaka. In Daakaka, adjectives can be distinguished from verbs by the presence of a copula *i* in predicative contexts. In the same contexts, stative verbs appear in their bare form (cf. von Prince 2015, pp. 47ff, 123ff).

(19)

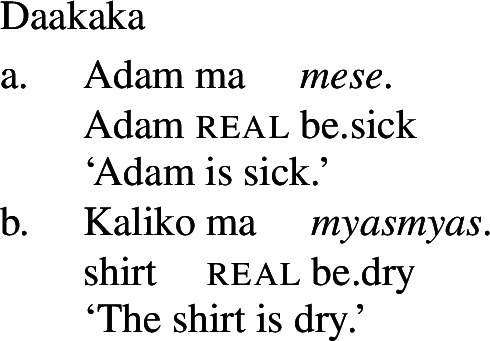

(20)

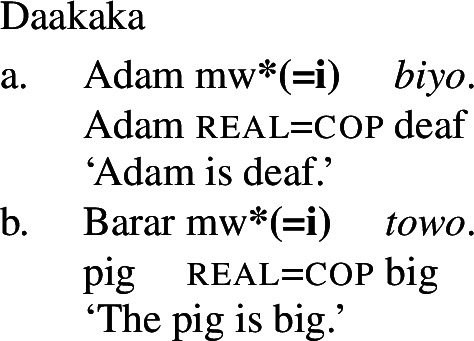

As a labile language, Daakaka permits stative VPs to undergo Inchoative Shift in the presence of event-selecting material. As (21) and (22) show, this applies both to VPs headed by stative verbs and to those containing adjectival phrases headed by the copula *i*.

(21)

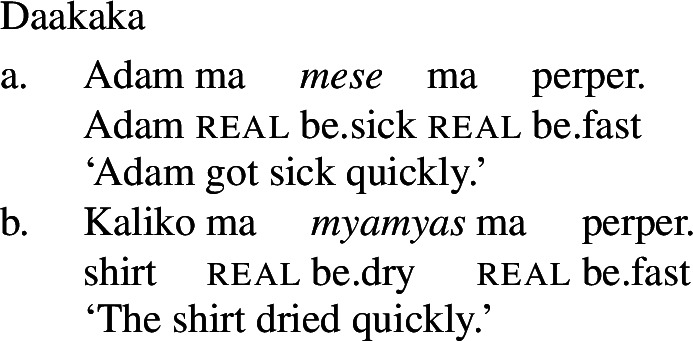

(22)

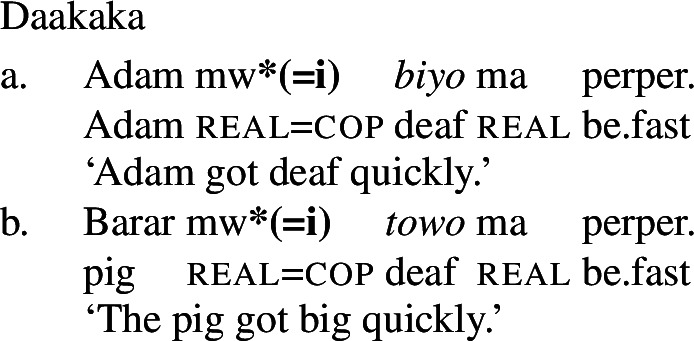

Given the verbal nature of copular phrases, it is at first blush difficult to see any difference between adjectival and verbal categories with respect to the application of Inchoative Shift. Fortunately, there are other contexts in which VPs and APs may appear, but, crucially, copulas may not. For example, VPs and APs may be nominalized, diagnosed by the presence of the article *an* following the nominalized phrase (cf. von Prince 2015, p. 107ff). APs are nominalized directly, with the presence of the copula rendering such nominalizations ungrammatical (24).

(23)

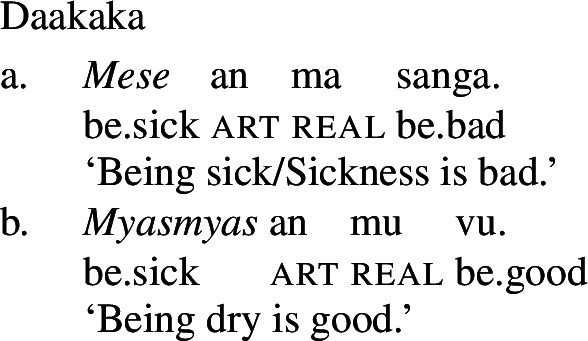

(24)

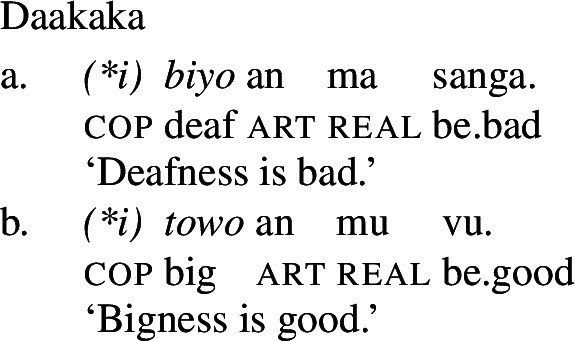

A nominalized stative VP may still undergo Inchoative Shift in the presence of a rate adverbial, as (25) shows.^7^ However, a nominalized AP *cannot*, and an attempt to modify such an AP with a rate adverb results in ungrammaticality, as (26) demonstrates.

(25)

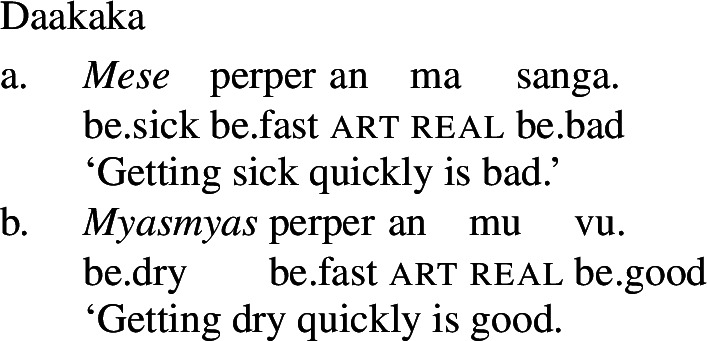

(26)

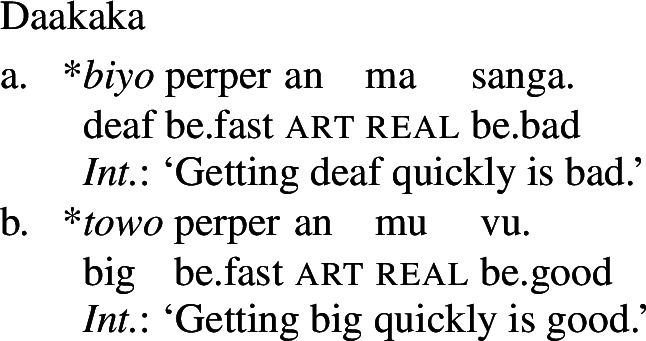

Since VPs headed by stative verbs may undergo Inchoative Shift when nominalized, it cannot be that Inchoative Shift cannot be applied within noun phrases. Rather, this evidence suggests that it is the presence of the copula, a verbal category, that results in the availability of Inchoative Shift with APs in the main clause. In contexts in which the copula is necessarily absent, like the nominalizations in (26), Inchoative Shift cannot apply to APs.

A similar categorial restriction has been described by Thornes (2003) for Northern Paiute (Numic), which, like Daakaka, exhibits distinct classes of verbal and adjectival stative lexemes. The two can be distinguished on the basis of the absence of nominalizing morphology on stative verbs in attributive contexts: while attributive adjectives like *undi* ‘tall’ directly combine with case morphology, stative verbs like *yuhu* ‘be.fat’ must be nominalized in attributive position.^8^

(27)
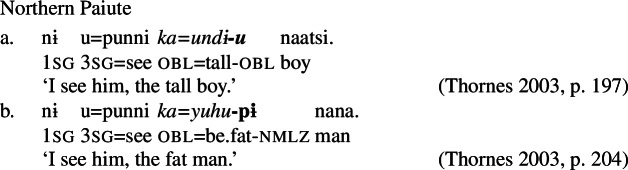

To express change-of-state meaning, adjectival lexemes must combine with a inchoative verb *ʔmani* ‘become’. In contrast, stative verbs are labile, and may express inchoative semantics by combining directly with the perfective aspect marker -*pi*.

(28)
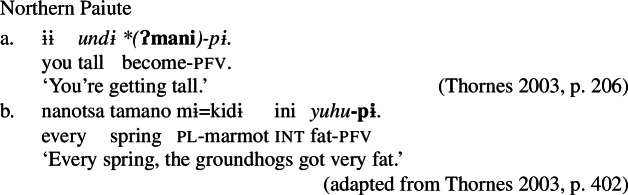

We thus find language-internal support for the restriction of Inchoative Shift to the meanings of verbal constituents, in addition to the aforementioned evidence from typological surveys.

Further to this discussion, we note that the restriction of type-shifting rules to particular categories is not unprecedented. For example, Partee’s (2002) ∃ and *ι* type-shifters are restricted to applying to NPs, though type-theoretically they could in principle apply to VPs and APs as well, at least in so far as these categories denote functions of type <e,t> at some point in a derivation.

### Durative adverbials

An additional prediction of this formulation of Inchoative Shift comes from its interaction with durational adverbials. In English, durational *for*-adverbials are ambiguous in the context of change-of-state predicates, and can modify one of two eventualities contributed by the predicate: the (result) state, giving rise to the *internal reading*), or the change/process event, giving rise to the *durative reading* (Dowty 1979; Piñón 1999). We illustrate the ambiguity in (29) below.

(29)
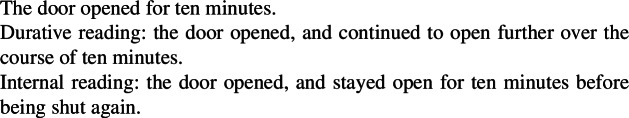

The same process/state ambiguity is observed in Mandarin with lexical change-of-state verbs, which lack corresponding stative construals, and thus always entail the existence of a change of state event.^9^ Here, bare post-verbal duration phrases give rise to both internal and durative readings (Zhang 2023); in (30), it is either the ship’s sinking or the state of the ship being sunk that lasted for three hours.

(30)
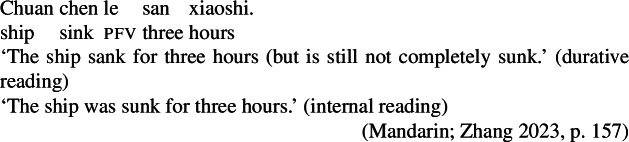

We can consider what our approach predicts for cases where a change-of-state predicate is derived by Inchoative Shift. Because durational adverbs can measure states just as well as events, they are not predicted to trigger Inchoative Shift. In other words, durational adverbials should only measure the duration of the state entailed by the stative predicate in a labile language like Mandarin, with no eventive construal possible without additional material requiring the presence of an eventive predicate. This first prediction is borne out: in (31), the temporal modifier *yi xiaoshi* ‘one hour’ measures the duration of Zhangsan’s state of being tall, but cannot measure the duration of the change of state, even with contextual support.

(31)
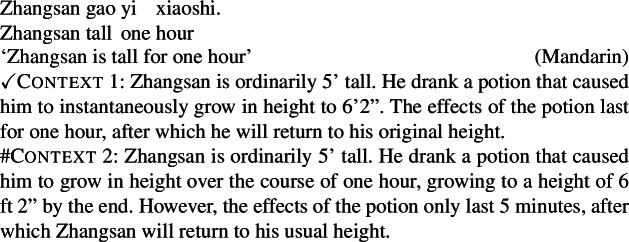

Our analysis makes a second, more subtle prediction as well, relating to the relative timing of VP-internal adverbial modification and Inchoative Shift triggered by VP-external aspectual marking. At the level of durational modification, a property concept verb like *gan* ‘dry’ or *gao* ‘tall’ simply denotes a predicate of states. Modification by a durational adverbial thus returns an internal reading. Only after composing with the perfective aspect marker *le* does the VP undergo type-shift to a predicate of events. This is to say, a sentence like (32) only has one attachment site for the durational adverbial, and thus can only have the structure in (33).

(32)

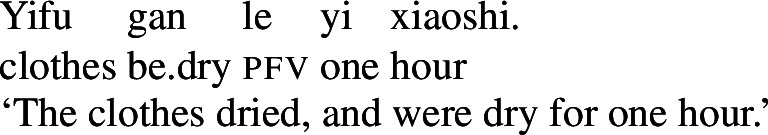

(33)

As a consequence of this, we predict a contrast between lexical change-of-state verbs like *chen* ‘sink’ and change-of-state inferences derived via Inchoative Shift, as with verbs like *gan* ‘dry’: while a durative reading of a durational modifier is available with the former class, we expect durative readings to be absent with the second class. In other words, Mandarin change-of-state predicates derived by Inchoative Shift should only ever have an internal reading when modified by durational adverbs.

This prediction is borne out: in Mandarin, the durative reading is unavailable with change-of-state predicates derived from stative VPs by Inchoative Shift. As such, only internal readings are attested with durational adverbials. For example, the only possible interpretation of (32), repeated below, is that the clothes were in a dry state for one hour; no durative reading, in which the clothes gradually dried over the course of an hour but may not be entirely dry, is available (Zhang 2023).

(34)
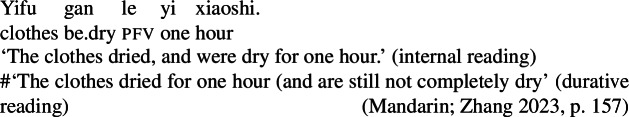

The absence of a durative reading of shifted VPs with durational adverbials in the context of Inchoative Shift follows from Inchoative Shift’s nature as a last-resort operation: the type-shift only applies to in order to repair clashes between types. Consequently the shifted meaning composes only with the expression it underwent the type-shift to compose with. An internal reading is available because a predicate of states is available for the durational phrase to compose with, but no event predicate is available for the durational phrase to modify, thereby ruling out the durative reading.^10^

## A Blocking Principle for Inchoative Shift

### Morphology vs. type-shifting

The type-shifting perspective on lability also lends itself to an explanation for *why* Inchoative Shift is available in labile languages like Tongan, Mandarin, and Daakaka, but not in languages falling into other derivational types, like Japanese and Korean. The starting point in the development of our account is the observation that the latter group of languages possesses overt morphology dedicated to the expression of change-of-state semantics, while labile languages lack such morphology. In essence, *the presence of a form expressing change-of-state meaning blocks the application of Inchoative Shift in a language.* This connection between the presence/absence of dedicated morphology and the (un)availability of Inchoative Shift is presented in tabular form below.

(35)
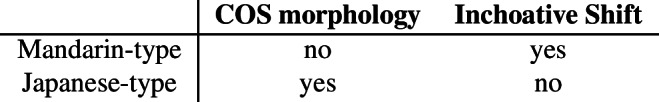

The idea that the existence of overt forms in a language for expressing certain meanings rules out the application in that language of type-shifting principles that would otherwise express those same meanings has much precedent in semantic theory. To give a particularly prominent example, in a seminal paper, Chierchia (1998) aims to explain the fact that some languages, such as Mandarin, permit bare noun phrases, which may have either definite or indefinite meanings, as shown by (36).

(36)
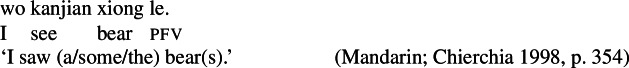

The essence of Chierchia’s explanation for this phenomenon is that the availability of definite and indefinite readings of bare nouns in Mandarin is a consequence of the fact that Mandarin lacks determiners that would otherwise express such meanings. In type-shifting terms, Chierchia proposes that Partee’s ∃ and *ι* type-shifters may apply to Mandarin noun phrases precisely because no form in the language exists that would otherwise express their meanings. Languages like English, on the other hand, make use of the articles *a* and *the* for the purpose of expressing the same meanings as the ∃ and *ι* type-shifters. As such, ∃ and *ι* cannot apply to English NPs, thus explaining the unacceptability of singular bare nouns in English, as seen in (37).

(37) I saw *(a/the) bear.

Chierchia formalizes the effect of overt expressions on the availability of covert type-shifting operations in terms of a *Blocking Principle*, which states that type-shifting is only permitted if no overt determiner does the same semantic work as a type-shifting operation.

(38)
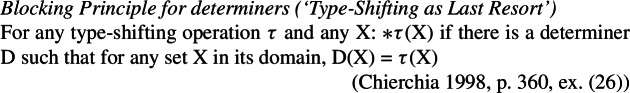

While Chierchia’s Blocking Principle is designed to apply to type-shifts expressible by determiners, it is plausible that it is a special case of a more general principle ruling out any type-shifting operation in languages with overt morphosyntactic means to express the same meaning as the type-shifter in question. We can thus extend Chierchia’s Blocking Principle to account for blocking effects with type-shifting outside of the nominal domain. Our first attempt at such a Generalized Blocking Principle is provided in (39).

(39)
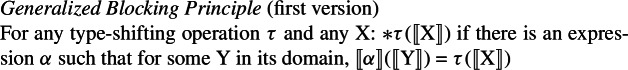

Change-of-state lability, and its absence in non-labile languages, can therefore be seen as part of a broader phenomenon concerning the inventory of type-shifting operations in language and the principles that constrain the availability of those operations cross-linguistically.

### Apparent counterexamples: periphrastic and morphological inchoatives

A certain amount of caution is necessary in properly formulating the Generalized Blocking Principle. In particular, our first attempt at formulating such a principle in (39) predicts that any overt means of expressing change-of-state semantics in a language will block Inchoative Shift in that language. This is a very strong prediction, and is thus easy to falsify along two independent lines. First, we find that the existence of *periphrastic* inchoative constructions in a language does not always block Inchoative Shift cross-linguistically. For example, Daakaka permits Inchoative Shift despite the option of using an inchoative verb, *me*, to express change of state (40) (cf. von Prince 2015, p. 356f). This is also true of Mandarin, which possesses an overt inchoative verb *bian* (41).

(40)

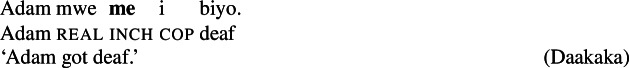

(41)
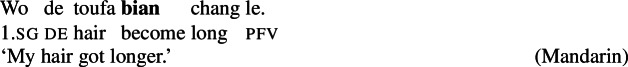

Yet despite the fact that periphrastic inchoatives do not prevent type-shifting by Inchoative Shift in Daakaka and Mandarin, such inchoatives do seem to block Inchoative Shift of copular clauses in languages like English, as can be seen in (42).

(42)
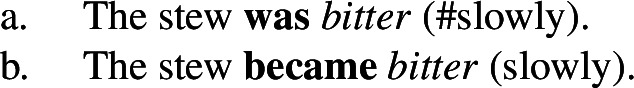

While this fact about English may appear to be unsurprising given the restriction of Inchoative Shift to verbal constituents, it does not actually fall out from the fact that adjectives in English are nonverbal. Recall that copular VPs in Daakaka can undergo Inchoative Shift. If copular phrases are verbal in English as well, then it is not immediately clear why Inchoative Shift should be ruled out, other than by an appeal to the presence of the periphrastic inchoative.

Second, even in languages that appear to have a morphological means of deriving change-of-state verbs from stative verbs, Inchoative Shift appears to be possible, at least in some environments, in contrast to our observations in languages like Japanese and Korean. For example, in the language isolate Wá⋅šiw (also spelled Washo), change-of-state verbs are derived from stative verbs by the suffix *-etiʔ* (Bochnak 2015, 2023).

(43)
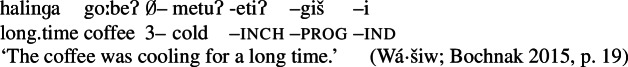

Nevertheless, Bochnak points out that the progressive marker -*giš* may compose directly with stative verbs, resulting in a change-of-state reading without *-etiʔ*.

(44)
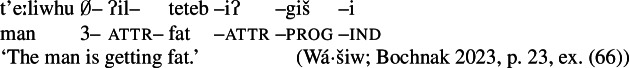

To explain this fact, Bochnak claims that the change-of-state interpretation in the absence of *-etiʔ* is derived via coercion. If we take coercion here to be Inchoative Shift, then (44) constitutes another apparent counterexample to the Generalized Blocking Principle. All else being equal, the Wá⋅šiw data, along with the data from periphrastic inchoatives above, present a problem for our explanation for the cross-linguistic distribution of state/change-of-state lability.

There are reasons to believe, however, that all else is not equal in the interaction between Inchoative Shift and the structure of inchoative constructions cross-linguistically. Focusing first on periphrastic inchoatives, upon closer inspection, English and Daakaka differ in the way that their periphrastic inchoative interacts with copular constructions. In English, inchoative verbs like *become* are in complementary distribution with the copula *be* and combine only with nonverbal predicates, as is clear in (45).

(45) Kim became (*be) deaf/a teacher.

Daakaka, however, behaves differently: the inchoative verb *me* only combines with verbal constituents (46), which makes the presence of the copula *i* obligatory in the context of adjectival predicates like *biyo* ‘deaf’.

(46)
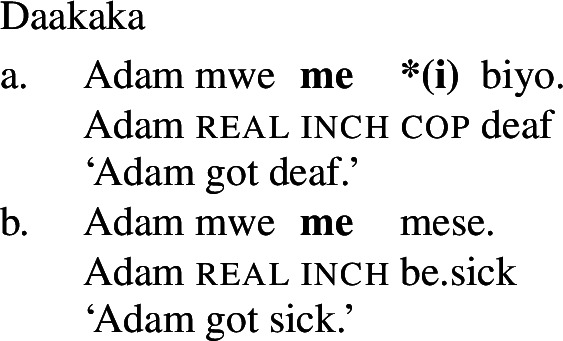

Periphrastic inchoatives therefore vary in their *structural complexity* across languages: in English, *become* and the copula *be* are in complementary distribution, and structures containing them are thus equally complex. In Daakaka, on the other hand, the inchoative construction *contains* the copular structure, and is thus demonstrably *more* complex than an equivalent sentence with only the copular structure.

A similar observation can be made about the inchoative in Wá⋅šiw. Wá⋅šiw stative verbs are morphologically complex: in addition to the root, many stative verbs contain one or more so-called “attributive” morphemes (Jacobsen 1964). Hanink and Koontz-Garboden (2021) argue that this attributive marking, particularly the morpheme *–iʔ*, serves to categorize the root as a verb, and introduces possessive semantics between the state the root introduces and the holder of the state.

(47)
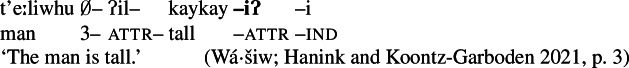

Crucially, the inchoative marker -*etiʔ* attaches to attributive-marked forms, rather than to the root, as (48) shows.^11^

(48)
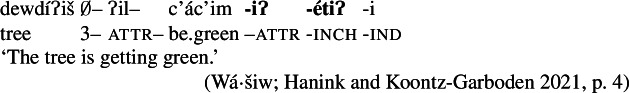

This shows that -*etiʔ* should not be analyzed as a verbalizer, but is instead much more analogous to the periphrastic inchoative in Daakaka by virtue of being strictly more complex than the stative verb upon which it is built. In this, Wá⋅šiw differs from Japanese, where stative and inchoative forms of property concept verbs are equipollently derived: while stative forms are marked by *-kar* in all forms except the present in (9-b),^12^ inchoative forms are marked by a different suffix, *-gar* or *-mar*, depending on root class. Crucially, the inchoative suffix does *not* attach to the stative form, but directly to the root (cf. Oseki 2017; Nakajima 2021). Stative and inchoative forms are thus equally complex.^13^

(49)
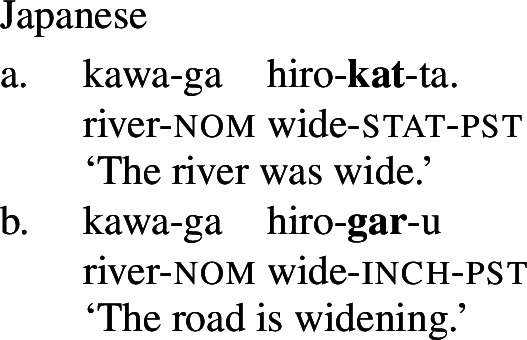

As a result, morphological inchoatives resemble periphrastic inchoatives as they can vary cross-linguistically in their morphosyntactic complexity.

### Structural alternatives and the Blocking Principle

The notion of structural complexity has played a major role in recent work in natural language semantics.^14^ In particular, Katzir (2007) and Fox and Katzir (2011) argue that the alternatives relevant to the computation of scalar implicatures are derived by operations of *replacement* and *deletion* on syntactic structures.^15^ Katzir defines the *structural complexity relation between parse trees* ≤ as in (50), with the set of *structural alternatives* to a parse tree *ϕ* defined in (51).

(50)

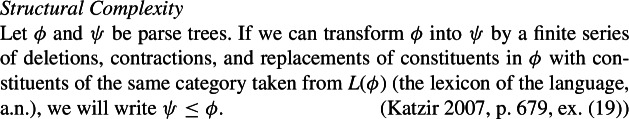

(51)
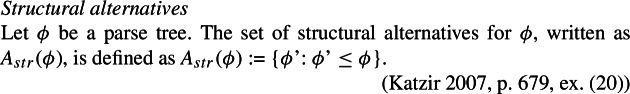

By the definition of structural complexity, any parse tree in the set of structural alternatives to *ϕ* has at most as many nodes as *ϕ*: in other words, all parse trees in *A*_*str*_(*ϕ*) are at *most* as complex as *ϕ* itself, and, due to the application of deletion operations, may be strictly *less* complex than *ϕ*. As a consequence, if a structure is strictly *more* complex than *ϕ*, it cannot serve as an alternative to *ϕ*. In the domain of scalar implicatures, this provides a solution to the *symmetry problem*, where sentences stronger than the assertion that are equally complex as the asserted proposition, but not those that are strictly more complex, may act as alternatives. For example, *some of the dogs are brown* competes with *all of the dogs are brown*, because *all* may replace *some* in an otherwise identical parse tree, but it does not compete with *some but not all of the dogs are brown*, which, though stronger than the assertion, is strictly more complex, and cannot be derived as a structural alternative.

Given our appeal to structural complexity in distinguishing English and Daakaka periphrastic inchoatives, we can make use of Katzir’s formalization of structural alternatives to modify our first attempt at a Generalized Blocking Principle for type-shifts to capture our intuition that it is precisely this difference between the complexity of periphrastic inchoatives and that of copular structures in a language that affects the availability of Inchoative Shift in that language. First, we will be more explicit about the structure of periphrastic inchoatives in English and Daakaka. Note that in English the inchoative verb *become* and the copula *be* embed nonverbal complements, such as an AP or an NP. We take this to indicate that inchoative and copula verbs are equivalent in structural complexity, and both inchoative and copular structures in English involve a VP headed by the inchoative verb/copula, with an AP or NP complement (52).

(52)
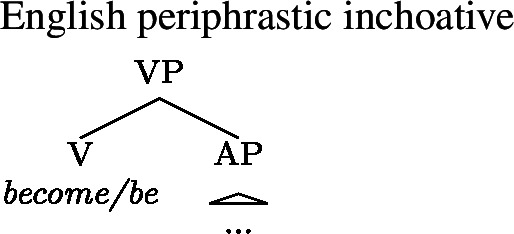

In Daakaka, on the other hand, the periphrastic inchoative verb *me* embeds stative verbal complements. This includes stative VPs headed by the copula *i*. Unlike English, Daakaka periphrastic inchoatives are thus structurally more complex than an equivalent sentence with the copula, as a copular structure is clearly embedded within the larger periphrastic inchoative structure. We thus posit the structure in (53) for Daakaka periphrastic inchoatives, in which the copular VP, headed by *i*, serves as complement to the inchoative verb *me*, itself the head of its own VP. The same analysis holds for Mandarin periphrastic inchoatives headed by *bian*, which takes a stative (property concept) VP as a complement, as well as for Wá⋅šiw -*etiʔ*, which demonstrably attaches to a verbal projection headed by the attributive marker -*iʔ*.

(53)
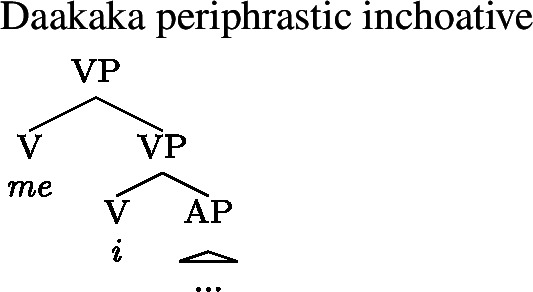

Next, we will refine our Generalized Blocking Principle from Sect. 3 so that it makes reference to structural alternatives. Our second, final version of the Blocking Principle is presented in (54).

(54) *Generalized Blocking Principle with structural alternatives*
  For any type-shifting operator *τ* and any X: ∗*τ*(〚X〛) if there is an expression Y such that Y ∈ *A*_*str*_(X) and 〚Y〛 = *τ*(〚X〛)

The structural properties of English and Daakaka periphrastic inchoatives interacts with (54) to derive the differential availability of Inchoative Shift in each language. First, note that the application of Inchoative Shift to a stative constituent has the same meaning as applying the meaning of an inchoative verb to that constituent. This satisfies the second part of the condition on the application of (54). The difference between English and Daakaka (and Mandarin) comes down to the first part of the condition. The structure of English periphrastic inchoatives in (52) is such that *become* and *be* occupy the same structural position. This means that a periphrastic inchoative can be derived from a copular VP by performing a substitution operation on the head of the VP, and therefore the former counts as a structural alternative to the latter. This satisfies both parts of (54), and for this reason Inchoative Shift is blocked with English copular constructions.

In Daakaka, on the other hand, the periphrastic inchoative *embeds* the copular VP. As the former is strictly *more* complex than the latter, it cannot be derived from the latter by a series of deletion and substitution operations. The periphrastic inchoative is thus *not* a structural alternative to the copular construction in Daakaka, and the Generalized Blocking Principle cannot apply. Inchoative Shift is thus correctly predicted to be available with Daakaka copular VPs, as desired.

Our revised Generalized Blocking Principle also accounts for the more basic contrast between labile languages like Daakaka and Mandarin and non-labile languages like Japanese and Korean. Recall that our original motivation for the Blocking Principle was the intuition that English and Japanese, but not Daakaka and Mandarin, possess dedicated morphology for the expression of change of state. Our proposal can account for the absence of Inchoative Shift in Japanese-like languages in one of two ways. The first way would be to adopt a lexicalist analysis, on which both stative verbs and change-of-state verbs are single V heads. On this approach, a VP headed by a change-of-state verb would count as a structural alternative to one headed by a stative verb, triggering the Generalized Blocking Principle and ruling out Inchoative Shift in Japanese. The second way would be to adopt a decompositional analysis along the lines of approaches within the Distributed Morphology framework (Harley 2008). On this style of analysis, both stative and change-of-state verbs are decomposed into an acategorial root and a little *v* head that serves to categorize that root as verbal. The difference lies in what *flavor* of *v* is used: *v*_be_ is stative, and denotes an identity function on predicates of states, while *v*_become_ is eventive, with inchoative semantics (Folli and Harley 2005).

(55)

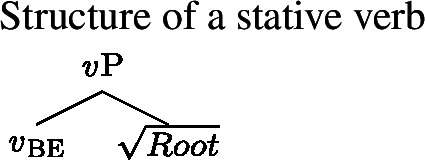

(56)
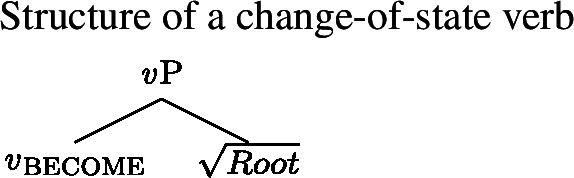

On this decompositional analysis, the structure corresponding to the change-of-state verb is still a structural alternative to that corresponding to the stative verb, for the same reason as in the analysis of English periphrastic inchoatives: the change of state *v*P can be derived from the stative one by substituting *v*_become_ for *v*_be_. Therefore, the Generalized Blocking Principle applies, and Inchoative Shift is ruled out on this analysis as well.

Regardless of which of the two analyses above one prefers, the ultimate theoretical proposal remains the same: Inchoative Shift is ruled out by the presence of a structural alternative to a stative VP with inchoative semantics. Likewise, the explanation for the availability of Inchoative Shift in labile languages is the same on both analyses: these languages lack change-of-state morphology (whether lexical or in the form of a *v*_become_), so no structural alternative to a stative VP exists that has the same meaning as the application of Inchoative Shift to that VP.

## Alternative analyses

In this section, we consider potential alternatives to our analysis. We begin with the possibility that apparent Inchoative Shift arises due to a null morpheme that introduces change-of-state semantics directly. We then consider Koontz-Garboden’s (2007) approach to state/change-of-state lability in Tongan couched in terms of coercion. In both of these cases, we show that our analysis as developed above is to be preferred by virtue of its superior predictions with respect to the phenomena under discussion.

### Lability as zero morphology

A straightforward alternative to our analysis would simply assert that all of these languages possess zero morphology that encodes change-of-state semantics. On our type-shifting approach, there is no morpheme, overt or covert, that introduces change-of-state semantics. The primary motivation for this assumption comes from the observation that change-of-state semantics always relies on additional material that selects for a dynamic eventuality, such as rate adverbials, progressive and perfect aspect, or certain restructuring verbs like *’osi* ‘finish’ in Tongan. While our type-shifting account naturally accounts for this co-variation, it is left unexplained under an approach that derives inchoative predicates from stative verbs with zero morphology, as we would expect the inchoative interpretation to be independently available regardless of the broader morphosyntactic or semantic environment of the verbal predicate (cf. Koontz-Garboden 2007 for further discussion).^16^ There do exist languages in which stative and inchoative forms are truly ambiguous, and thus allow both stative and inchoative interpretations even in the absence of material that forces an inchoative meaning. Jamul Tiipay is such a language, as can be seen in (57) (Miller 2001; cf. also adjectival stative/verbal inchoative pairs like *narrow*/*to narrow* in English).

(57)
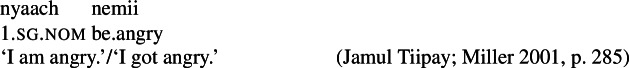

Due to the absence of material that would require Inchoative Shift in the inchoative form, we might assume a zero morpheme that combines with the stative form and introduces change-of-state semantics to account for cases like in (57) (an alternative being lexical ambiguity). Importantly, though, this is not the case for the languages we have considered throughout this paper, and a key point of our analysis is that change-of-state semantics is *not* freely available. This fact is readily explained by our type-shifting analysis, but not on an analysis that generalizes zero morphology to all cases of zero derivation of the inchoative. On our view, then, the source of zero inchoativization varies across languages: in some languages, such as Tongan, Daakaka, and Mandarin, change-of-state semantics arises from Inchoative Shift, while in others, such as Jamul Tiipay, an adequate analysis could well appeal to null derivational morphology.

### Lability as coercion: Koontz-Garboden (2007)

In a previous analysis of lability as it occurs in Tongan, Koontz-Garboden (2007) argues that change-of-state inferences with stative property concept lexemes are derived through *coercion*. What this amounts to on Koontz-Garboden’s formulation, building on the approach of Pustejovsky (1995), is that if two syntactic elements have meanings that are inconsistent with one another compositionally, the meaning of one will alter in order to accommodate the other. In this case, in combination with event-selecting material, a stative verb is coerced into a change-of-state predicate, allowing it to compose with material it would otherwise be incompatible with.

*Prima facie*, Koontz-Garboden’s coercion analysis appears to be very similar to our analysis in terms of Inchoative Shift: while Koontz-Garboden does not explicitly define the coercion process he proposes, it is clear that the variety of coercion he has in mind delivers change-of-state semantics, just like Inchoative Shift. Also like our Inchoative Shift rule, coercion applies as a repair process to render otherwise semantically incompatible expressions compatible compositionally.

From a broader perspective, however, our proposal ultimately differs significantly from Koontz-Garboden’s. First of all, Koontz-Garboden’s coercion analysis does not readily explain the complementary distribution of state/change-of-state lability and the presence of change-of-state morphology within a language. On our approach, this follows from the independently motivated idea that type-shifting operations are blocked by the existence of overt expressions that do the same semantic work as the type-shift. While one could in principle develop a similar proposal for coercion operators, we are not aware of any independent precedent for such an idea in the literature on coercion, unlike in the type-shifting literature, and note that Koontz-Garboden proposed no such account himself. Our proposal thus presents a step forward from Koontz-Garboden’s work.

Relatedly, Koontz-Garboden’s coercion analysis is not sensitive to the structural complexity of alternative sentences. It thus offers no explanation for the distinction between morphological and periphrastic inchoatives with respect to blocking coercion. While Koontz-Garboden does not discuss periphrastic inchoatives in his paper, if Tongan possesses such inchoatives, it is not clear from his proposal why coercion would be necessary, if a form expressing change-of-state semantics is available in the language. The approach we advocate makes very specific predictions in this domain, and is further able to explain why periphrastic inchoatives do not block Inchoative Shift in some languages, such as Daakaka and Mandarin, but does in languages like English.

Finally, while one *could* read Koontz-Garboden’s proposal as similar to our own type-shifting analysis, one need not do so; a coercion analysis simply says that when there is a mismatch between two expressions, it is resolved. This leaves open the possibility that it could be resolved in a variety of different ways. That is to say, there is nothing in a coercion analysis that rules out the possibility of, for example, the *rate adverbial* shifting to a meaning consistent with stativity (while still obeying the monotonicity principle; cf. Koontz-Garboden 2007), rather than the attested shift of the verb’s meaning. Our own proposal is much more specific: it is the VP’s meaning that undergoes a shift. This allows for a straightforward explanation of the sort of meaning that arises across the grammatical constructions in which it does arise.

Taken as a whole, our analysis improves on Koontz-Garboden’s original coercion approach, as it makes correct predictions about the cross-linguistic distribution of state/change-of-state lability and its interaction with other inchoative expressions in different languages.

## Additional issues

Before proceeding to the conclusion, we briefly discuss potential confounds that may at first blush appear to undermine certain aspects of our proposal. First, we consider the distinction between *total and partial change*, and apparent cross-linguistic variation with respect to the kind of change that Inchoative Shift expresses. We then discuss independent inceptive readings associated with the rate adverbial *quickly* and its translational equivalents in other languages, and show that the availability of such readings does not undermine our type-shifting analysis. Finally, we consider Matthewson et al.’s (2015) analysis in which the perfect itself introduces change-of-state semantics.

### Total and partial change

Change of state comes in at least two forms. Following Bochnak’s (2023) terminology, we refer to these as *total* and *partial* change. *Total change* describes a change from lacking to having some scalar property to a degree that exceeds the contextual standard at the end of the change, i.e., entailing positive semantics. *Partial change*, on the other hand, describes a change in some scalar property without necessarily exceeding the contextual standard at the end of the change, i.e., not entailing positive semantics. In English, total change is observed with the verb *become* in combination with an NP or an AP headed by a positive form adjective.

(58) The river became wide.
  ⇝ The river’s width exceeds the contextual standard for width at the end of the change of state.

Partial change, in contrast, can be expressed by degree achievements based on open-scale adjectives, or by the combination of *become* with a comparative adjective.

(59)
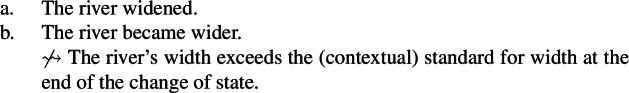

Recall that our Inchoative Shift rule is formulated in terms of a become operator, along the lines of Dowty (1979) and Beavers and Koontz-Garboden (2020). Applied to a stative VP with positive semantics, this predicts that such VPs should involve total change, rather than partial change. This prediction is clearly borne out in Daakaka; the application of Inchoative Shift to a positive VP gives rise to a total change reading. This can be seen in (60), where an attempt to deny that Angela is tall after the change of state results in a contradiction.

(60)
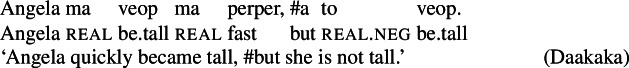

When we shift our attention to Mandarin, however, Inchoative Shift of bare stative VPs does not appear to be associated with positive semantics; rather, shifted VPs are associated with entailments of *partial* change; as (61) shows, they are true in a context in which the individual undergoing the change does not exceed the standard for the scalar property encoded in the verb. In contrast to Daakaka, Mandarin type-shifted VPs thus seem to have the kind of meaning associated with English degree achievements (Zhang 2023).

(61)
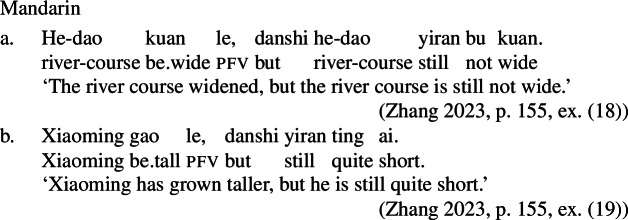

This state of affairs may lead one to conclude that we need a different Inchoative Shift rule for Mandarin, one that delivers entailments of partial change. One way to accomplish this is by having Mandarin’s Inchoative Shift make use of *measure of change functions*, developed by Kennedy and Levin (2008) to account for the semantics of degree achievements in English. An attempt at formulating this variety of Inchoative Shift is given in (62).

(62) *Inchoative Shift* (degree-achievement type)
  For a verbal constituent V such that 〚V〛 is of type <s,d>, shift(〚V〛) = *λ*e.〚V$]\!\!]_{\Delta}$(e) > pos(〚V$]\!\!]_{\Delta}$), where 〚V$]\!\!]_{\Delta}$(e) = 〚V〛(fin(e)) – 〚V〛(init(e)) and fin(e) and init(e) are the final and initial states of e, respectively.

Adopting different rules for Daakaka and Mandarin may be premature, however. First, note that shifted VPs in Mandarin can combine with a *bi* ‘than’-phrase, which introduces the standard of comparison. Note, in particular, that the verb remains bare, with no additional comparative morphology beyond the *bi*-phrase.

(63)
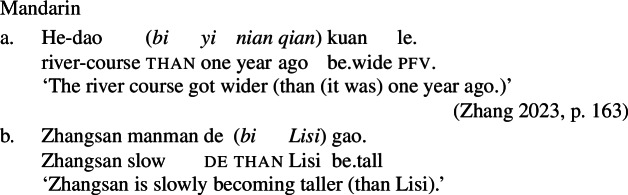

More generally, bare stative VPs in Mandarin are independently known to possess comparative readings in the absence of the degree morpheme *hen* (Grano 2011; Zhang 2021; Sun 2022; Gong and Coppock 2024). In many cases, the comparative reading is the only natural one; for example, (64-a) is interpreted as comparative even in the absence of the standard phrase headed by *bi*. However, the positive is possible in some circumstances, such as in sentences with small clauses like (64-b), and, more generally, in sentences involving contrastive focus, as in (64-c) (Zhang 2021; Sun 2022, *pace* Grano 2011).

(64)
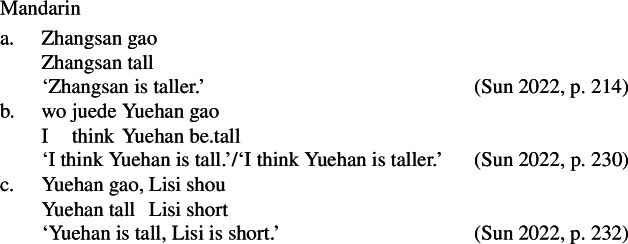

Because of the independent availability of comparative semantics in these cases, we can account for the presence of partial change readings with Inchoative Shift in Mandarin without baking partial change into our type-shifting rule. In particular, we can account for these readings by having Inchoative Shift, with the same semantics it has in Daakaka, apply to a stative VP with comparative semantics. One possible analysis would be to treat the meaning of this phrase as a predicate of states that exceed a contextually specified degree, building off the approach to comparatives in Wellwood (2015).^17^

(65) 〚Zhangsan gao$]\!\!]^{c} = \lambda $s.holder(s) = zhangsan ∧ tall(s) ∧ *μ*(s) > d_*c*_

In combination with *le*, our original Inchoative Shift rule is triggered, delivering (66).

(66) *λ*e.∃s[become(e,s) ∧ ∃s[holder(s) = zhangsan ∧ tall(s) ∧ *μ*(s) > d_*c*_]]

Upon existential closure of the event variable e, (66) will be true iff prior to e Zhangsan’s height did not exceed the contextually provided degree, but did exceed it after e. In other words, Zhangsan became taller than he was previously, the right result. This shows that Inchoative Shift formulated in terms of a become operator based on total change can, in combination with an independently available comparative semantics for the stative VP, capture apparent partial change readings.

Partial change readings with Inchoative Shift arise in languages other than Mandarin as well. For example, in Daakaka, a partial change reading comes about in the context of shifted comparative stative VPs, overtly derived by the comparative verb *save* ‘exceed’ (cf. von Prince 2015, p. 354).

(67)
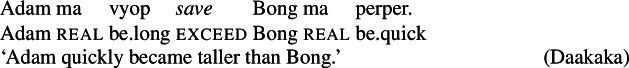

We can thus maintain a uniform approach to Inchoative Shift as involving total change via the become operator, with apparent partial change readings arising when it applies to a VP with independently available comparative semantics.

### Revisiting rate adverbials

So far, we have assumed that rate adverbials like *quickly* are incompatible with stative verb phrases in languages where Inchoative Shift is blocked by respective morphology. Yet, many languages, including English, actually allow a combination of a stative expression and *quickly*, giving rise to an inceptive reading that looks much like a case of Inchoative Shift (Creswell 1978; Pustejovsky 1992; Rawlins 2013; Koev 2017).

(68)
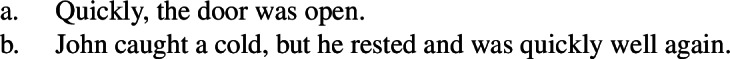

The availability of this reading with *quickly* in English appears to challenge our Blocking Principle, which predicts the unavailability of Inchoative Shift in English. One alternative to our analysis, then, is that Inchoative Shift is generally available, and there is no blocking to be explained.

There are two reasons to reject this alternative as an analysis of labile languages in favor of our own analysis, however. First of all, inceptive readings with *quickly* and other *adverbs of space and time* have an independent explanation. In the literature, such readings have been referred to as *narrative readings* (Rawlins 2013; Koev 2017). The narrative reading is not restricted to stative predicates, but also occurs with dynamic predicates, particularly achievement predicates like *notice*. As achievements occur instantaneously, the narrative reading amounts to measuring the amount of time between the beginning of the eventuality and some salient prior point in time in the given discourse, as can be seen in (69). The stative examples in (68) exhibit the same reading involving a short span of time between a prior state and the current one.

(69) The professor walked in and Kim quickly noticed him.

The narrative reading can be derived from a general semantics for *quickly*, as shown in Rawlins (2013) and Koev (2017) (see also Creswell 1978 for an early approach). Especially important for our purposes, though, is the fact that the narrative reading is highly restricted: unlike *quickly*, *slowly* lacks a narrative reading in English (Koev 2017), and similar constraints have been noted for other languages as well (cf. Maienborn 2005 and Schäfer 2013 on German; Hopperdietzel 2024 on Samoan).^18^

(70)
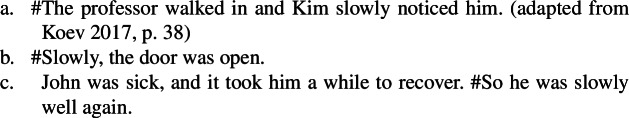

Due to the restriction of narrative readings to *quickly*, we can see clearly that an analysis of English and other languages like it in terms of Inchoative Shift is not supported. However, for labile languages, our analysis predicts that both *quickly* and *slowly* trigger Inchoative Shift, and thus we expect that no contrast should exist between *quickly*-type and *slowly*-type adverbs. This is borne out, as illustrated for Mandarin in (71), and for Daakaka in (72).

(71)

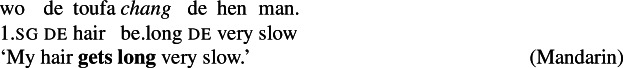

(72)
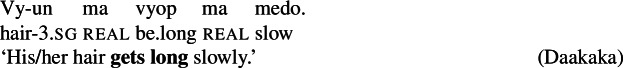

As for the second issue, as noted above, Inchoative Shift occurs in more environments than just in the context of rate adverbs. While one could, in principle, adopt an analysis of the fact in (72) on which *man* ‘slowly’ simply permits narrative readings in the same way as *kuai* ‘quickly’, such an analysis misses the broader generalization in Mandarin that event-selecting material generally gives rise to change-of-state readings with stative VPs in Mandarin. Our analysis in terms of Inchoative Shift, on the other hand, is able to account for this generalization without invoking a large number of otherwise unmotivated lexical ambiguities.

### Lability in the context of the perfect

Matthewson et al. (2015) develop an analysis of state/change-of-state lability in the context of perfect constructions in Niuean, which are quite similar to those in closely related Tongan, as discussed in Sect. 4.2 above. On their analysis, change-of-state meaning is built into the perfect marker itself (*kua* in Niuean). On the surface, this would seem to obviate the need for a type-shifting analysis, since on such an analysis there is overt morphology, albeit coupled with the semantics of the perfect, which serves to derive a change of state from a stative predicate.^19^ As Matthewson et al. (2015, p. 28) show, however, change-of-state meaning can also arise in Niuean in the context of rate adverbials, and crucially without the perfect marking *kua*, just as in Tongan. They therefore suggest that change-of-state meaning can be introduced in Niuean not only with the perfect, but also by a coercion process like Koontz-Garboden’s, a process that we recast here as Inchoative Shift (Matthewson et al. 2015, p. 32).

We are not in a position to argue against Matthewson et al.’s analysis. As they themselves say, “[t]here is no reason why there should not be more than one source of inchoativity in a language, and indeed there often is” (Matthewson et al. 2015, p. 28), and there is nothing in our analysis here that would preclude this being the case, with change of state introduced by the perfect morphology when it can be, and via type-shift otherwise. A careful analysis on a language-by-language basis is necessary to establish whether Inchoative Shift is at play in the introduction of inchoative meaning. Ultimately, because this is a matter that boils down to a question about the right analysis of the perfect in Niuean, rather than a question about our type-shifting operation—which is consistent with Matthewson et al’s analysis—we leave it to the side here, but believe it merits highlighting as an area for future work.

## Conclusion

In this paper, we have proposed a novel analysis of state/change-of-state lability as a variety of type-shifting, Inchoative Shift. We have argued that this perspective readily explains the morphological shape and semantic properties of state/change-of-state lability cross-linguistically, including the fact that it is not freely available, but only resolves type-mismatches when stative VPs combine with material that requires a predicate of dynamic eventualities. On our approach, Inchoative Shift is tied to a Blocking Principle that constrains, and thereby explains, its cross-linguistic distribution: Inchoative Shift is only possible in languages without separate change-of-state morphology. Our formulation of this Blocking Principle appeals to structural alternatives, and thereby explains the fact that Inchoative Shift is not incompatible with the presence of periphrastic inchoatives in a language, and, more broadly, forges a connection between constraints on the availability of type-shifting operations cross-linguistically and the more general role that alternatives play in natural language semantics and pragmatics. Our proposal improves on previous analyses by situating the phenomenon in a broader empirical and theoretical context, with clear predictions about its distribution.

In closing, we discuss how our proposal fits more broadly into the type-shifting literature, and how the inventory of type-shifting operations can be restricted. It has at times been asserted in the literature that type-shifting operations should be restricted to logical or “topic-neutral” operations that do not add lexical meaning (Van Benthem et al. 1986; Kratzer 2005). Kratzer (2005) in particular refers to operations like her Causative Shift and, by extension, our Inchoative Shift, which are not topic-neutral, as having a “blemish”, and argues they should be avoided. On the other hand, positing similar operations that add certain kinds of “lexical” meaning to semantic representations has proved useful in other domains. As an example, Sawada and Grano (2011) present evidence for an operation of *Scale Shift*, which converts gradable adjectives into comparatives in the presence of a measure phrase.^20^

(73) Scale Shift (Sawada and Grano 2011, p. 216, ex. (72))
  *λ*g.$\lambda \mathrm{x.g}^{\uparrow}_{s}$(x), where s is a contextually determined standard and g^↑^ is the difference function derived from g (Kennedy and Levin 2008)

Evidence for an operation like Scale Shift comes from Japanese, where gradable adjectives (or stative verbs) possess comparative meanings in the context of measure phrases. Crucially, in the absence of a measure phrase, no comparative meaning is available, and the positive interpretation obtains instead, as in (74) (from Sawada and Grano 2011, p. 218, ex. (75)).

(74)
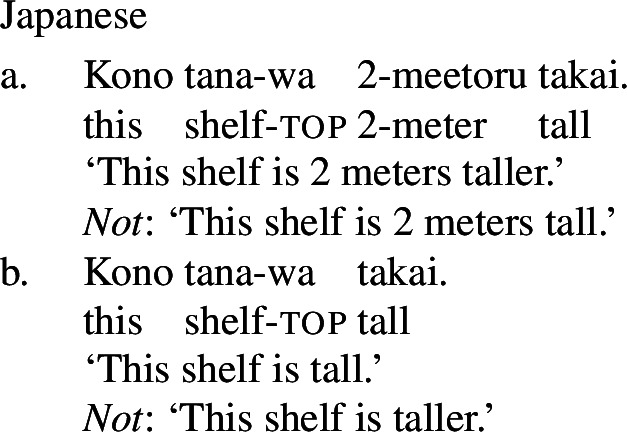

On Sawada and Grano’s approach, as on our own, Scale Shift is triggered only when there is a clash between the meaning of the two expressions, in this case the meaning of the measure phrase and that of the gradable adjective. Moreover, Sawada and Grano appeal to Chierchia’s (1998) Blocking Principle to explain the absence of similar effects in other languages, making crucial reference to the absence of comparative morphology on Japanese adjectives. Our approach thus finds some precedent in this work on Japanese comparative meanings derived by a last-resort operation coupled with a Blocking Principle.

This said, the question of what constrains the range of type-shifting operations available to the grammars of natural languages remains an important one. We suggest that one plausible constraint on possible meaning-adding type-shifting operations consonant with our approach is that type-shifts express meanings that are *overtly expressed by functional heads in some languages*. In other words, operations like Inchoative Shift and Scale Shift are available to natural languages because they have corresponding functional heads (*v* and comp) that instantiate them. This rules out many conceivable type-shifting operations, such as one that adds, say, both comparative and change-of-state meaning at the same time, on the assumption that no functional head encodes such a meaning. It also eliminates many implausible type-shifting operations, such as one that, say, requires that an event happen on a Tuesday, since, again, no functional head expresses such a specific meaning. In combination with the last-resort nature of type-shifting and the Blocking Principle, we expect to find a restricted set of type-shifting operations, which may add certain kinds of meanings to the basic meaning of an expression as long as the additional meaning is i) compositionally motivated and ii) not otherwise expressed by another expression of the language with the same structural complexity. Whether this set of constraints is enough to constrain the set of type-shifters is ultimately, we think, an empirical question, that can only be answered with further detailed cross-linguistic research. What we have proposed here takes an initial step in the development of this larger research program.

## Footnotes

1 The term *labile* originates in Haspelmath (1993), though in the context of causative-inchoative verb pairs. Our use of this term follows that in Beavers et al. (2021) and differs from Haspelmath’s in referring to COS-stative lexeme pairs.

2 See Sect. 4 for a detailed argument against a zero derivation analysis of state/change-of-state lability.

3 We assume here that states and events are of different types, s in the case of states and v in the case of events. One can imagine alternatives: for instance, if both states and events are considered to be of a more general type of eventualities, one would instead distinguish them as subsorts, much as it is possible to consider eventualities and entities as subsorts of a broader type of individuals. Our rule would then be more properly considered a *sort-shifting* operation, and would shift a stative predicate to an eventive one in the context of material that is only defined for predicates of eventualities of the event subsort.

4 become operators were proposed as early as Dowty (1979), where they were defined in terms of temporal intervals rather than events. Our own analysis draws on that of Beavers and Koontz-Garboden (2020), where become is defined as a relation between an event and a state.

5 The analysis of the rate adverb in (13) should be seen as a simplification of a more sophisticated analysis, such as that developed by Rawlins (2013) or Koev (2017). The choice of analysis does not affect the point here, as both analyses require the argument of a rate adverb to be a predicate of dynamic events.

6 In Daakaka, perfect/completive aspect is expressed via a serial verb construction including the non-inital verb *mo nok* ‘be finished’ (cf. von Prince 2015, p. 340).

7 As an anonymous reviewer notes, if we take Inchoative Shift to be applicable only to verbal constituents, then it should not be possible to apply it after nominalization. One possibility is that nominalization takes place in the syntax, with a nominalizing head taking a verbal or adjectival constituent as complement. On this approach, Inchoative Shift would apply to the verbal layer of the structure embedded under the nominal layer. Such an analysis is consistent with our general architectural assumptions and with the Daakaka data presented here.

8 The perfective marker *i* also functions as a nominalizer in Northern Paiute. See Thornes (2003, p. 104ff) for a detailed discussion on its (historical) relation to noun class markers.

9 The argument in this section focuses on Mandarin for two reasons. First, previous work by Zhang (2023) explicitly showed that Mandarin change-of-state predicates derived from stative verbs lack durative readings with durational modifiers, so independent evidence was available in the literature on Mandarin to cite. Second, of the labile languages for which we worked directly with native speaker consultants, Mandarin was the only one that possessed a reliable contrast between equivalents of *for*- and *in*-phrases. In our previous fieldwork on Daakaka, for instance, no direct equivalents for *for*- and *in*-phrases could be found, so data relying on this contrast was not collected.

10 An anonymous reviewer asks what implications this has for the analysis of eventive verbs like *chen* ‘sink,’ which, on some analyses, may nevertheless be decomposed into distinct eventive and stative structural layers. The stative layer could, given its type, undergo Inchoative Shift, as long as its category is verbal. For one, we follow Zhang (2023) in the treatment of *chen* ‘sink’ as an eventive predicate, with no underlying stative analysis. Even on a decompositional approach, if the eventive layer is always present, as seems to be the case with this verb and others like it, an aspectual head like *le* will never be in a local enough relationship with it to trigger Inchoative Shift. Furthermore, given the independent presence of an eventive component to the meaning of *chen*, we will expect the existence of a durative reading regardless of whether Inchoative Shift may apply to a putative stative constituent.

11 This holds as well for other expressions that, according to Bochnak (2023), trigger change-of-state coercion in Wá⋅šiw, such as *mamaʔ* ‘finish’.

12 In the present tense, statives appear without *-kar* and are simply marked by *-i*, which marks both present tense and category. Categorization and tense-marking are thus fused in the present tense.

13 There is an ongoing debate about the categorial status of stative PCs lexemes in Japanese (cf. Nishiyama 1999; Menon 2016; Baker 2003 for detailed discussion). Here, we adopt the spirit of Menon (2016) and assume that *-kar* is a stative verbalizer, similar to the attribuive suffix *–iʔ* in Wá⋅šiw.

14 A somewhat similar idea appears in the Distributed Morphology tradition, in which competition between forms is locally restricted to the same syntactic node (Embick and Marantz 2008). One could propose something along these lines by saying that Inchoative Shift is not blocked in Daakaka because it is not in competition with morphosyntactic inchoativization in this language, whereas it is in English. This would require, however, that Inchoative Shift be conceptualized as syntactically projected, e.g., in a syntactic terminal node. Our formulation of Inchoative Shift as a type-shifting rule does not assume this. We therefore adopt the Katzirian approach developed in the main text.

15 See also Rett (2014, 2020), who argues for a similar role for structural complexity in the domain of manner implicatures.

16 A potential alternative morphosyntactic account could assume that a zero inchoativizing morpheme is only inserted in contexts where it is needed, similar to what has been proposed by Grano (2011) for the obligatory presence of *hen* ‘very’ when stative PCs, which for Grano are of adjectival category, function as the main predicate in Mandarin.

We note three potential complications for such a morphosyntactic version of Inchoative Shift: first, morphosyntactic last-resort operations are typically triggered by morphosyntactic incompatibility, e.g., T selecting for a complement of verbal category in Grano’s analysis, whereas Inchoative Shift is triggered by semantic incompatibility. Second, we are not aware of any language in which morphological marking accompanies broader morphosyntactic environments that trigger Inchoative Shift in other languages. Without such morphological evidence, such an analysis is not falsifiable. Relatedly, it would raise the question of why in so many languages the morpheme takes the same phonological shape, namely null. Given the arbitrariness of the sign, this would be a spectacular coincidence.

17 We remain agnostic with respect to whether the comparative is introduced by a null morpheme, as in Grano (2011), or is inherent in the verb, along the lines of Zhang (2023).

18 An anonymous reviewer points out that German permits apparent change-of-state coercion of stative predicates with *langsam* ‘slow’. We provide the reviewer’s example below.

We do not consider this a case of Inchoative Shift in German. As Schäfer (2002) discusses, *langsam* can give rise to a reading in which a large amount of time has elapsed between the onset of an event or state and a prior reference time. Although this reading is not as readily available for *langsam* ‘slowly’ as for *schnell* ‘quickly’ (Schäfer 2013), it is a narrative reading, which is the reading on display in (69). The independence of this reading can be highlighted by the combination of two rate adverbs within the same sentence.

In the absence of a reference time, such examples are infelicitous. This contrasts with the examples in (71) and (72), in which it is the change of state that occurs slowly, and no prior reference time is needed for the examples to be felicitous.

19 Matthewson et al. (2015) also point out that Niuean *kua* differs from its Tongan equivalent *kuo* in lacking existential and universal readings of the perfect with stative predicates, and thus, unlike in Tongan, the presence of the perfect marker in Niuean always results in a change-of-state reading with stative predicates.

20 Technically speaking, Sawada and Grano characterize Scale Shift as a *coercion* operation, triggered not to repair type clashes, but by a need to compose with a measure function whose underlying scale possesses a minimum. The ideas behind their approach can most likely be translated into one more akin to our own analysis, on which the type of the adjective/stative verb must be adjusted in order to compose with a measure phrase. For example, if, as argued by Wellwood (2015), adjectives/stative verbs in Japanese denote not measure functions, but predicates of states, composition with a measure phrase may fail in the absence of a function from degrees, which the comparative plausibly is on a Wellwood-style approach. Scale Shift can then easily be recast in terms of a type-shifting operation permitting stative verbs to compose with degree-denoting material.
